# Molecular Characterization of *Cryptosporidium* spp. in Children from Mexico

**DOI:** 10.1371/journal.pone.0096128

**Published:** 2014-04-22

**Authors:** Olivia Valenzuela, Mariana González-Díaz, Adriana Garibay-Escobar, Alexel Burgara-Estrella, Manuel Cano, María Durazo, Rosa M. Bernal, Jesús Hernandez, Lihua Xiao

**Affiliations:** 1 Departamento de Ciencias Químico Biológicas, Universidad de Sonora, Hermosillo, Sonora, México; 2 Servicio de Infectología, Hospital Infantil del Estado de Sonora, Hermosillo, Sonora, México; 3 Laboratorio de Parasitología y Micología, Hospital Infantil de México Federico Gómez, México Distrito Federal, México; 4 Laboratorio de Inmunologia, Centro de Investigación en Alimentación y Desarrollo A. C., Hermosillo, Sonora, México; 5 Centers for Disease Control and Prevention, Atlanta, Georgia, United States of America; Cornell University, United States of America

## Abstract

Cryptosporidiosis is a parasitic disease caused by *Cryptosporidium* spp. In immunocompetent individuals, it usually causes an acute and self-limited diarrhea; in infants, infection with *Cryptosporidium* spp. can cause malnutrition and growth retardation, and declined cognitive ability. In this study, we described for the first time the distribution of *C. parvum* and *C. hominis* subtypes in 12 children in Mexico by sequence characterization of the 60-kDa glycoprotein (GP60) gene of *Cryptosporidium*. Altogether, 7 subtypes belonging to 4 subtype families of *C. hominis* (Ia, Ib, Id and Ie) and 1 subtype family of *C. parvum* (IIa) were detected, including IaA14R3, IaA15R3, IbA10G2, IdA17, IeA11G3T3, IIaA15G2R1 and IIaA16G1R1. The frequency of the subtype families and subtypes in the samples analyzed in this study differed from what was observed in other countries.

## Introduction

Cryptosporidiosis is a parasitic disease caused by *Cryptosporidium* spp. These parasites belong to the phylum Apicomplexa and are intracellular protozoa that infect mammals, birds, reptiles and amphibians. They are cosmopolitan, mostly affecting people with immunodeficiency and, in some cases, can be deadly. In immunocompetent individuals, *Cryptosporidium* causes acute diarrhea, usually self-limited, nausea, vomiting, loss of appetite, weight loss and fever [1]–[5]. In infants, infection with *Cryptosporidium* spp. may cause malnutrition and permanently affect growth, resulting in a functional decline in physical fitness and cognitive ability [6]–[10]. Parasite transmission occurs via the fecal-oral route through the ingestion of contaminated water or food, person-person contact, or animal contact [11]–[13].

Human cryptosporidiosis is mainly caused by the species *Cryptosporidium hominis* and *Cryptosporidium parvum* [14]; the distribution of these species varies temporally and geographically [15]. The former mainly infects humans (>70% of human cryptosporidiosis is caused by *C. hominis* in most countries) [16], while the latter infects humans as well as domestic and wild ruminants [17], [18]. A few other species have been reported in humans, including *C. meleagridis*, *C. felis*, *C. canis*, *C. cuniculus, C. ubiquitum*, *C. viatorum, C. suis*, *C. muris,* and *C. andersoni* [16], [19]–[22].

The identification of oocysts in stool using the Ziehl-Neelsen modified stain commonly known as Kinyoun [23]–[25] is the most commonly used method in diagnosing cryptosporidiosis. However, it does not allow for the identification of species, which are morphologically indistinguishable but genetically distinct. To determine *Cryptosporidium* species, restriction fragment length polymorphism (RFLP) analysis is often performed based on the gene of the small subunit rRNA (SSU rRNA) or the *Cryptosporidium* oocyte wall protein (COWP) [14], [26]–[33]. To differentiate subtype families of *Cryptosporidium*, the gene encoding the 60-kDa glycoprotein (GP60) is employed [34], [35]. In the case of *C. hominis*, 6 subtype families have been identified (Ia, Ib, Id, Ie, If and Ig), with at least 78 subtypes. In *C. parvum,* 10 subtype families (IIa, IIb, IIc, IId, IIe, IIf, IIh, IIi, IIj, IIk) have been identified, with at least 78 subtypes [16], [34]–[40]. The majority of subtype families infect both humans and animals (especially ruminants); however, the IIc (formerly Ic) subtype family has only been isolated in humans [38].

In Mexico, there have been very few studies of human cryptosporidiosis [41]–[43], and none of these determined *Cryptosporidium* species and subtypes. The objective of this work was to characterize *Cryptosporidium* spp. identified in stool samples of children in Mexico.

## Methodology

### Ethics Statement

The protocol of this project was approved by the Ethics Committee of Hospital Infantil del Estado de Sonora. Informed consent was obtained from each *Cryptosporidium*-infected patient who voluntarily participated after a clear explanation of the research objectives. Parents or guardians signed consent on behalf of the children enrolled in this study. Samples obtained from Hospital Infantil Federico Gómez in Mexico City were originally submitted for parasitic analysis, positive samples to *Cryptosporidium* included in this study were kindly provided by Dr. Rosa Maria Bernal. The stool samples of infants were carriers of *Cryptosporidium* spp., diagnosed on microscopic observation of oocysts [44]. The inclusion criteria of participation was: *Cryptosporidium* spp. infected patients regardless of age, gender, with or without clinical symptoms and patients who consented to the study, whereas the exclusion criteria were those who were not *Cryptosporidium* spp infected and who did not give their consent to participate in the study. Clinical data were obtained from patient’s medical record with patient’s consent and permission from health authorities. Fecal samples were stored at 4°C for further analysis.

### Stool Samples

Stool samples were analyzed from 12 children (2 girls and 10 boys) from 7 months to 14 years of age who were carriers of *Cryptosporidium* spp. as diagnosed on microscopic observation of oocysts [44]. Four samples were obtained from the Hospital Infantil del Estado de Sonora, and 8 were obtained from the Hospital Infantil Federico Gómez in Mexico City (Table 1). They were diagnosed as *Cryptosporidium*-positive by modified Kinyoun method described by Henriksen [44]. The cases included in this study occurred from October 2010 to July 2013 (Table 1).

**Table 1:** Demographics of cryptosporidiosis cases from México and genotypes *Cryptosporidium* isolates in this study.

SampleID	Age	Gender	Clinical symptom	Geographicorigin	Collection date	DNA origin	COWPsequencingspecie/genotype	18S rRNAPCR-RFLPspecie/genotype	GP60sequencingspecie/genotype	GP60sequencingsubtype family and subtype
S1	9 Y	Male	HIV, CO SM lll, AGE, S	HS	June 2013	Feces	*C. parvum*	*C. parvum*	*C. parvum*	IIa A15G2R1
S2	14 M	Male	P, MM, AGE	HS	June 2013	Feces	*C. hominis*	*C. hominis*	*C. hominis*	Ie A11G3T3
S3	8 M	Male	AGE	HS	July 2013	Feces	*C. hominis*	*C. hominis*	*C. hominis*	Ie A11G3T3
S4	7 M	Female	AGE, P, MM	HS	July 2013	Feces	*C. hominis*	*C. hominis*	*C. hominis*	Ie A11G3T3
M2	12 M	Male	NA	DF	October 2010	Conatin	*C. hominis*	*C. hominis*	*C. hominis*	Ia A15R3
M3	14 Y	Male	CRI, TK	DF	April 2011	Conatin	*C. hominis*	*C. hominis*	*C. hominis*	Ia A14R3
M4	4 Y	Female	SD, CG	DF	May 2011	Conatin	*C. hominis*	*C. hominis*	*C. hominis*	Ia A14R3
M5	5 Y	Male	HIV, M	DF	July 2011	Conatin	*C. parvum*	*C. parvum*	*C. parvum*	IIa A16G1R1
M6	4 Y	Male	HIV, NA	DF	September 2011	Conatin	*C. hominis*	*C. hominis*	*C. hominis*	Ia A14R3
M8	9 M	Male	NA	DF	December 2011	Conatin	*C. hominis*	*C. hominis*	*C. hominis*	Ib A10G2
M12	3 Y	Male	PT, AGE	DF	August 2012	Conatin	*C. hominis*	*C. hominis*	*C. hominis*	Id A17
M13	2 Y	Male	NA	DF	October 2012	Conatin	*C. hominis*	*C. hominis*	*C. hominis*	Ia A15R3

All 12 samples were from urban areas in México: HS, Hermosillo, Sonora, DF, Distrito Federal. Y, years. M, months. SM, severe malnutrition. MM, moderate malnutrition. AGE, acute gastroenteritis. CRI, chronic renal illness. SD, Syndrome Down. M, mentally handicapped. P, pneumonia. CO, candidiasis oral. S, sepsis. CG, congenital glaucoma. PT, postransplant. HIV, HIV-positive. TK, transplant of kidney. NA, not available.

### Oocyst Concentration

1 to 2 g of feces was homogenized with 10 ml of physiological saline solution (PSS). The suspension was filtered through cheesecloth into a 15-ml conical tube and centrifuged at 2,000 rpm for 1 minute. The supernatant was decanted, and the pellet was resuspended with 15 ml of PSS. The process was repeated 2 to 3 times until the supernatant was clear. Then, 10 ml of 5% formalin was added to the supernatant, mixed and allowed to stand 10 minutes. Next, 5 ml of ethyl acetate was added, and then the tube was capped and shaken vigorously for 30 seconds, uncovered carefully and centrifuged at 1,500 rpm for 2 minutes. A wooden applicator was inserted into the tube to release the border of the layers. Carefully, the layers were decanted without disturbing the sediment. The sediment was resuspended in 3 ml of 0.2 N NaOH. The mixture was incubated at 37°C for 30 minutes, washed twice with PSS and centrifuged at 2,000 rpm. The PSS was removed with a pipette and, the number of oocysts in the sediment was determined by staining of a smear of the sediment using the Kinyoun method described above.

### DNA Extraction

DNA extraction was performed directly from ∼200 µl of stool or ∼200 µl of the oocyst concentrate using the QIAamp DNA Stoll Mini Kit (QIAGEN Inc, Valencia, CA) following the recommendation of the supplier after 5 cycles of freezing and thawing (−70°C to boiling) of the oocysts. The extracted DNA obtained was stored at −20°C until further processing. As positive controls, we used DNA preparations (one each) from *C. parvum* and *C. hominis* from HIV patients in Peru, previously identified by PCR-RFLP analysis of the SSU rRNA gene and DNA sequencing of the gp60 gene [45].

### Molecular Characterization of *Cryptosporidium*

The molecular characterization of *Cryptosporidium* spp. was conducted by nested PCR analyses of 3 molecular markers: the small subunit rRNA (SSU rRNA) [21], COWP gene and GP60 [38], generating products that were of 826 to 864, 540 and 350 bp, respectively. To determine the species of *Cryptosporidium*, the nested PCR product of the 18S rRNA gene was digested using the *Vsp*I restriction enzyme (Promega, USA) [21]. To identify subtype families and subtypes of *Cryptosporidium*, PCR products of the GP60 gene were sequenced and subtype families were named as proposed by Strong et al., [35]. Each species of *Cryptosporidium* identified was assigned a Roman numeral; *C. hominis* was assigned I and *C. parvum* II. After indicating the species, the subtype family was identified with a lower case letter. For *C. hominis* the subtype families included Ia, Ib, Id, Ie, If and Ig; for *C. parvum* the subtype families included IIa, IIb, IIc, IId, IIe, IIf, lih, IIi, IIj, IIk. Subtypes within each subtype family were named according to the nomenclature proposed by Sulaiman [34], depending on the trinucleotide (TCA, TCG, and TCT) encoding the amino acid serine. Each time these sequences were repeated, they were assigned the capital letters A, G, and T, respectively. For *C. parvum* subtype family IIa, Sulaiman [46] used the letter R for the number of sequence ACATCA after the trinucleotide repeats, with R1 (one copy of TCAACA) for most of the IIa subtypes. Nucleotide GP60 sequences of *Cryptosporidium* obtained in this study have been deposited in the GenBank under accession nos. KJ460362, KJ460363, KJ460364, KJ460365, KJ460366, KJ460367, KJ460368, KJ460369, KJ460370, KJ460371, KJ460372, KJ460373.

## Results

### Age and Gender of Children with Cryptosporidiosis

Of the 12 children included in the study, 83% were boys (10/12) and 17% were girls (2/12). Of the children infected with *C. hominis*, 75% (9/12) were 0 to 4 years of age. The only 2 cases of *C. parvum* were boys, ages 5 and 9 years old, who were also diagnosed with HIV (Table 1).

### Species of *Cryptosporidium* Identified

**Figure 1:**
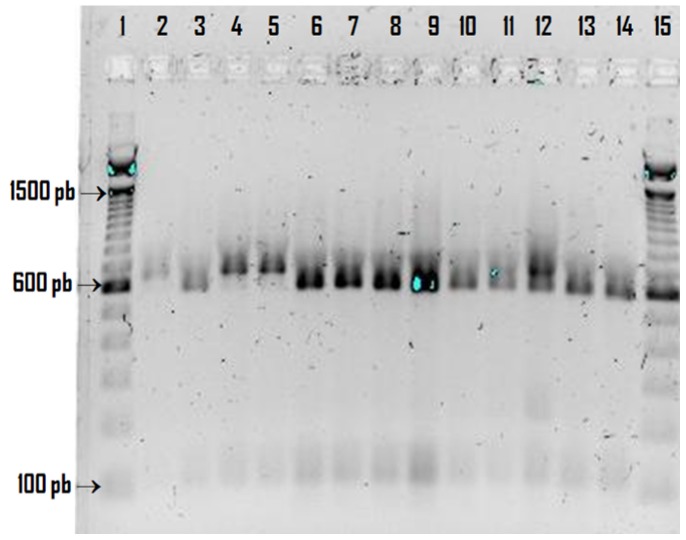
Results of *Vsp1* restriction digestion of the SSU rRNA gene of *Cryptosporidium* from México. Lane 1 and 15∶100-pb marker; lane 2: *C. parvum* control; lane 3: *C. hominis* control; lane 4: sample S1; lane 5: sample M5; lane 6: sample M3; lane 7: sample M4; lane 8: M2; lane 9: sample M13; lane 10: sample M8; lane 11: sample M12; lane 12: sample S2; lane 13: sample S3; lane 14: sample S4.

**Figure 2:**
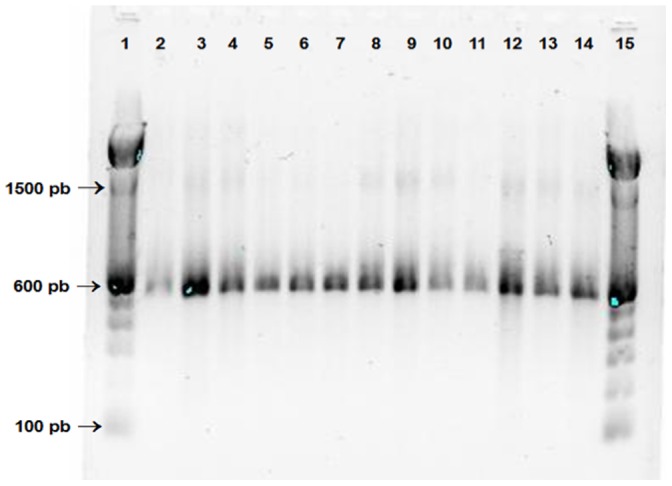
Results of the nested PCR of COWP gene of *Cryptosporidium* from México. Lane 1 and 15∶100-pb marker; lane 2: *C. parvum* control; lane 3: *C. hominis* control; lane 4: sample S1; lane 5: sample M5; lane 6: sample M3; lane 7: sample M4; lane 8: M2; lane 9: sample M13; lane 10: sample M8; lane 11: sample M12; lane 12: sample S2; lane 13: sample S3; lane 14: sample S4.

After determining the presence of *Cryptosporidium* using the Kinyoun method, the SSU rRNA gene was amplified and nested PCR products were digested with the restriction enzyme *Vspl,* producing the characteristic bands of *C. hominis* (102/104 bp and 561 bp) in 10 children (2 girls and 8 boys) and *C. parvum* (102/104 bp and 628 bp) in 2 boys (Figure 1), this result was confirmed by sequence analysis of the nested PCR products. The COWP gene was amplified and nested PCR products was confirmed by sequence analysis (Figure 2).

### Subtypes of *Cryptosporidium* Identified

Analysis of the GP60 gene sequences identified four subtype families in *C. hominis*: Ia (5/10), Ib (1/10), Id (1/10) and Ie (3/10) (Table 1). Within the Ia, we identified 2 subtypes: IaA15R3 (2/10) and IaA14R3 (3/10). For the Ib, Id and Ie, we identified only 1 subtype of each of these alleles: IbA10G2 (1/10), IdA17 (1/10) and IeA11G3T3 (3/10) (Table 1). For *C. parvum,* we detected the presence of only IIa subtype family, with 2 subtypes: IIaA15G2R1 (1/2) and IIaA16G1R1 (1/2).

## Discussion

In this work, we determined the species, subtype families, and subtypes of *Cryptosporidium* in stool samples in the state of Sonora and Mexico City. Of the 12 cases of cryptosporidiosis included in this study, we identified *C. hominis* in 10 cases and *C. parvum* in 2 cases. Our results are consistent with findings in developing countries where *C. hominis* is considered the predominant species in humans [47]–[49]. Despite the small number of *Cryptosporidium*-positive specimens in this study, we identified all 4 common subtype families of GP60 in *C. hominis*, including Ia, Ib, Id and Ie. Previously, Ib was the most frequently identified *C. hominis* subtype family [36], although in this study Ia and Ie were identified in 8 of 10 *C. hominis* samples. In this study, subtype IbA10G2 was only identified in one 9-month-old child, although it is the most common Ib subtype (88.5% within subtype Ib). The IbA10G2 subtype is considered the most common cause of outbreaks of waterborne cryptosporidiosis [16], [36], [50], [51], and has been identified in humans in Africa [39], Asia [34], Australia [50], [52], and in cattle in South American [16], [36], [50], [53].

The Id subtype, which was only identified in a 3-year-old child, was IdA17. There have been a few reports of this subtype isolated in humans in Australia (Western Region), Netherlands and Kenya (Nairobi) [54], [55]. In contrast, the IeA11G3T3 is the most prevalent Ie subtype [36]. In this study, IeA11G3T3 was one of the most prevalent and was identified in 3 patients in Sonora. These results agreed with the data by other researchers in India (Kolkata) [56], United Kingdom [57], US [58], Australia [52], Kuwait [34], Ecuador, Pakistan and Uganda [37], [56] and no reports in animals. In this study, 2 subtypes of family subtype Ia were identified: IaA15R3 (in 2 patients) and IaA14R3 (in 3 patients).

Most of the subtypes found in this study have been previously reported in humans in various countries, except IaA15R3 subtype, which has only been reported once by Hadfield in 2011 in a patient in the United Kingdom (GenBank HQ149032) [57].

In this study, 3 children participated in the study were diagnosed with HIV; unfortunately one of the children died. Two of the three children were infected with *C. parvum*; the child who died had IIaA15G2R1, and another child had the IIaA16G1R1 (Table 1). The two subtypes have been identified in farm animals [59]–[62] –[62– 65]. In addition, IIaA15G2R1 e has been reported as the most common subtype in HIV+ patients in Malaysia [66], and is commonly seen in humans in other countries such as Australia [50], Egypt [67] and the Netherlands [63]. The IIaA16G1R1 subtype has also been identified in humans in Slovenia [65] and the US [68,69]. The subtype family IIa is the most common *C. parvum* (57.8%) and is the second most frequently reported subtype family in humans (25.5%), with a global distribution (26 countries) [36].

In conclusion, in this work, *C. hominis* was the predominant species in 12 *Cryptosporidium*-positive children analyzed in Mexico. The frequency of the *C. hominis* subtypes identified in this study appear to be different from what was reported in other areas of the world. However, additional studies with a larger sample size in multiple states are needed to determine the subtypes of *C. hominis* and *C. parvum* in the country, better understand the transmission of cryptosporidiosis in humans, and assess the role of zoonotic transmission in cryptosporidiosis epidemiology.
